# Predictive value of red blood cell parameters for sepsis during chemotherapy-induced neutropenia in children with acute lymphoblastic leukemia based on propensity score matching

**DOI:** 10.3389/fped.2026.1795475

**Published:** 2026-04-10

**Authors:** Chuncan Wu, Chuan Tian, Liuhua Liao, Lili Liu, Zhonglv Ye, Min Chen, Weijun Huang, Qiping Yang, Xiang Lan

**Affiliations:** 1 Department of Pediatrics, Affiliated Hospital of Guangdong Medical University, Zhanjiang, China; 2 Department of Pediatrics, Huizhou Central People’s Hospital, Huizhou, China

**Keywords:** acute lymphoblastic leukemia, chemotherapy induced neutropenia, propensity score matching, red blood cell parameters, sepsis

## Abstract

**Objective:**

To investigate the predictive efficacy and clinical application value of red blood cell (RBC) parameters (hemoglobin and red blood cell distribution width) and their combined model for sepsis during chemotherapy-induced neutropenia (CIN) in children with acute lymphoblastic leukemia (ALL), based on propensity score matching (PSM) to eliminate confounding bias between groups.

**Methods:**

A retrospective cohort design was adopted. A total of 264 children with ALL who received induction remission chemotherapy according to the South China Children's Leukemia Group-ALL-2016 (SCCLG-ALL-2016) protocol and developed CIN between January 2021 and December 2024 in the Affiliated Hospital of Guangdong Medical University and Huizhou Central People's Hospital were included. The patients were divided into a sepsis group and a CIN group according to clinical outcomes. PSM was used to balance baseline characteristics between groups. Univariate and multivariate logistic regression models were used to screen independent influencing factors for sepsis. Receiver operating characteristic (ROC) curves, net reclassification improvement (NRI), and integrated discrimination improvement (IDI) were used to evaluate the predictive efficacy and incremental value of single indicators and the combined model. Decision curve analysis (DCA) was applied to assess clinical net benefit, and Kaplan–Meier curves were plotted to analyze the cumulative incidence of sepsis under different RDW quartiles.

**Results:**

A total of 108 patients were included after PSM matching (75 in the CIN group and 33 in the sepsis group). The RDW levels and pediatric Sequential Organ Failure Assessment (pSOFA) scores in the sepsis group were significantly higher than those in the CIN group, while Hb, RBC, and Hct levels were significantly lower (*P* < 0.05). Multivariate logistic regression analysis showed that RDW (OR = 1.424, 95% CI: 1.150–1.764, *P* = 0.001) and pSOFA score (OR = 1.613, 95% CI: 1.102–2.361, *P* = 0.014) were independent risk factors for sepsis, while Hb (OR = 0.974, 95% CI: 0.943–1.006, *P* = 0.113) was a protective factor. ROC curve analysis showed that the AUC of the combined model (Hb + RDW) for predicting sepsis was 0.812, which was significantly superior to single Hb (AUC = 0.672), RDW (AUC = 0.701), and pSOFA score (AUC = 0.682). Incremental value analysis showed that compared with single RDW, the IDI of the combined model was 0.068 (*P* = 0.003). DCA confirmed that the combined model provided better clinical net benefit within the threshold range of 10%–60%. Log-rank test indicated that the cumulative incidence of sepsis in the high RDW level group (Q4) was significantly increased (*P* = 0.024).

**Conclusions:**

RBC parameters (RDW, Hb) are independent predictors of sepsis in children with ALL during CIN. The combined prediction model constructed by Hb and RDW can effectively capture early signals of microcirculatory dysfunction, with predictive efficacy and clinical benefit superior to single indicators.

## 1 Introduction

Acute lymphoblastic leukemia (ALL) is the most common hematological malignancy in childhood, with an incidence of approximately 3–4 per 100,000 (1), accounting for about 25% of all pediatric malignancies (2). With the optimization of risk-stratified treatment strategies, the 5-year disease-free survival rate has improved to 70%–90% (3). However, high-intensity combination chemotherapy inevitably leads to severe myelosuppression and immune impairment (4, 5). Chemotherapy-induced neutropenia (CIN) is a common complication during induction remission and consolidation therapy (6). Due to the loss of the innate immune barrier mediated by neutrophils, susceptibility to opportunistic pathogens such as bacteria and fungi increases, potentially progressing to life-threatening sepsis (7). Epidemiological data indicate that sepsis progresses rapidly in children with ALL during chemotherapy; without early recognition and timely intervention, the mortality rate can reach 10%–30% (8). Traditionally, clinical practice relies on inflammatory markers like procalcitonin or C-reactive protein (CRP) for early warning. However, during the myelosuppression phase in children with ALL, the extreme scarcity of absolute neutrophil count (ANC) hinders the release of key inflammatory cytokines like interleukin-6 (IL-6). This delays the hepatic synthesis of acute-phase proteins such as CRP, resulting in an “immune silence” phenomenon where marker levels do not match the infection severity (9). Meanwhile, the widespread use of glucocorticoids during early infection can inhibit inflammatory protein transcription, further causing false-negative results and missing the optimal intervention window. Recently, red blood cell (RBC) parameters, as fundamental indices in routine blood tests, have garnered attention for their predictive value in infection pathophysiology. Studies suggest RBCs are not only oxygen carriers but also sensitive sensors of inflammatory storms and oxidative stress. In the early stages of sepsis, released pro-inflammatory mediators inhibit erythropoietin (EPO) receptor activity and interfere with normal erythroid maturation in the bone marrow, leading to loss of RBC volume uniformity. Simultaneously, pathogen-induced severe oxidative stress directly damages RBC membrane cytoskeletal stability, reducing deformability and increasing fragmentation (10, 11). Compared to specific biomarkers requiring separate blood sampling, RBC parameters—as part of routine blood counts—offer the unique advantage of high-frequency dynamic monitoring. For children with ALL requiring strict bone marrow function monitoring, these indices may possess superior early warning efficacy.

However, retrospective cohort studies investigating the predictive value of indicators for sepsis are often limited by the inherent defects of non-randomized data. Previous studies often lacked statistical control for potential confounding factors, leading to significant differences in baseline characteristics between groups and making it difficult to rule out selection bias (12). For instance, uneven distribution of covariates like admission season and chemotherapy phase between sepsis and non-sepsis groups may mask or exaggerate the true association between RBC parameter abnormalities and sepsis, thereby weakening the reliability of conclusions. To address these methodological limitations, propensity score matching (PSM), a statistical method based on the “counterfactual framework,” is widely used to correct for confounding factors in non-randomized studies (13). Its core advantage lies in compressing multidimensional complex covariates into a single propensity score and matching individuals with similar scores to maximize the balance of baseline differences, simulating a “randomization” effect in observational studies (14, 15). Currently, studies evaluating the predictive value of RBC parameters for sepsis in children with ALL during CIN after controlling for potential confounders using PSM remain scarce. Therefore, this study aims to use retrospective cohort data and apply PSM to eliminate baseline imbalances, investigating the independent predictive efficacy of RBC parameters for sepsis in children with ALL during CIN, with the goal of providing evidence-based medical grounds for early clinical warning.

## 2 Materials and methods

### 2.1 Study population

This retrospective cohort study was conducted involving children with ALL who were diagnosed and treated with the South China Children's Leukemia Group-Acute Lymphoblastic Leukemia-2016 (SCCLG-ALL-2016) protocol at the Affiliated Hospital of Guangdong Medical University and Huizhou Central People's Hospital between January 2021 and December 2024. The inclusion criteria were as follows: (1) diagnosis of ALL meeting the criteria of the SCCLG-ALL-2016 protocol and confirmed by comprehensive classification involving bone marrow cell morphology, immunology, cytogenetics, and molecular biology; (2) age < 18 years; (3) occurrence of chemotherapy-induced neutropenia (CIN), defined as an absolute neutrophil count (ANC) < 0.5 × 10⁹/L, during the first induction remission chemotherapy (VDLD regimen: vincristine, daunorubicin, L-asparaginase, and dexamethasone); and (4) complete clinical medical records. The exclusion criteria were as follows: (1) presence of other malignancies, congenital immunodeficiency, or autoimmune diseases; (2) severe dysfunction of vital organs (heart, liver, or kidney) or severe malnutrition prior to chemotherapy; (3) history of hematopoietic stem cell transplantation; (4) receipt of red blood cell suspension transfusion within 2 weeks prior to the onset of CIN or diagnosis of sepsis; and (5) history of iron deficiency anemia, thalassemia, or other non-chemotherapy-related hematological disorders affecting baseline red blood cell morphology; (6) patients who met the Sepsis-3 diagnostic criteria concurrently at the onset of CIN or within 24 h of the baseline laboratory sampling, to eliminate incorporation bias and ensure a strict predictive timeline.

The enrolled patients were divided into a sepsis group and a CIN group based on clinical outcomes during the CIN period. The diagnosis of sepsis followed the *Third* International Consensus Definitions for Sepsis and Septic Shock (Sepsis-3) (16), defined as life-threatening organ dysfunction caused by a dysregulated host response to infection. This study protocol was reviewed and approved by the Medical Ethics Committees of the Affiliated Hospital of Guangdong Medical University and Huizhou Central People's Hospital (Approval Nos. PJ2021-005 and ky112022004).

### 2.2 Diagnostic criteria and group definitions

Definition of chemotherapy-induced neutropenia (CIN): According to the Guideline for the Management of Fever and Neutropenia in Children with Cancer and Hematopoietic Stem-Cell Transplantation Recipients: 2017 Update (17), CIN was defined as an ANC < 0.5 × 10⁹/L after chemotherapy. In this study, the date when the ANC first dropped to 0.5 × 10⁹/L was designated as the onset date of CIN.

Diagnosis of sepsis: In this study, an increase in the pediatric Sequential Organ Failure Assessment (pSOFA) score of ≥2 points from baseline was used as the clinical diagnostic criterion for sepsis.

### 2.3 Data collection and laboratory measurements

#### 2.3.1 Clinical and general data collection

Clinical baseline data of the enrolled children were retrospectively collected from the hospital's electronic medical record system using a standardized case report form. The data collection primarily covered three dimensions: (1) Demographic and general characteristics: including age, gender, body mass index (BMI), number of hospitalizations, and year of discharge; (2) Disease characteristics: including leukemia immunophenotype (e.g., B-cell or T-cell type), clinical stage, and risk stratification; (3) Environmental and temporal factors: the season of admission was detailed to exclude the impact of seasonal variations in infection rates; and (4) Baseline organ function assessment indicators: physiological, biochemical, and physical sign parameters required to calculate the initial pediatric Sequential Organ Failure Assessment (pSOFA) score were collected synchronously on the onset date of CIN, including respiratory function (oxygenation index), hepatic and renal function (total bilirubin, serum creatinine), cardiovascular function (mean arterial pressure), and neurological assessment (Glasgow Coma Scale).

#### 2.3.2 Laboratory measurements

All laboratory indicators were measured within 24 h after the confirmation of CIN. Venous blood samples (2 mL) were collected by professional nursing staff under strict aseptic conditions into anticoagulant vacuum tubes containing dipotassium ethylenediaminetetraacetate (EDTA-K2). The samples were gently inverted for mixing and transported to the Department of Laboratory Medicine within 2 h for analysis using an automated hematology analyzer (Sysmex XN-3000). This study focused on extracting and recording red blood cell parameters, including red blood cell count (RBC), hemoglobin (Hb), hematocrit (Hct), mean corpuscular volume (MCV), mean corpuscular hemoglobin (MCH), mean corpuscular hemoglobin concentration (MCHC), and red blood cell distribution width (RDW), as well as white blood cell count (WBC), absolute neutrophil count (ANC), and platelet count (PLT).

### 2.4 VDLD chemotherapy regimen

The VDLD chemotherapy regimen consisted of the following: prednisone was administered at 60 mg/m²/day on days 1–7; dexamethasone was administered at 6 mg/m²/day on days 8–28, and the dose was tapered and discontinued starting from day 29; vincristine was administered intravenously at 1.5 mg/m² on days 8, 15, 22, and 29; daunorubicin was administered at 30 mg/m², with the standard-risk group receiving 2 doses (on days 8 and 15) and the intermediate- and high-risk groups receiving 4 doses (on days 8, 15, 22, and 29); and pegaspargase was injected intramuscularly at 2,500 IU/m² on days 9 and 23, for a total of 2 doses.

### 2.5 Sample size calculation

The sample size estimation for this study was based on the prevalence estimation formula for cohort studies. Referring to relevant literature (7), the probability of sepsis occurrence during CIN in children with ALL is approximately 16%(*p* = 0.16). The confidence level was set at 95% $\left(Z_{\alpha /2}=1.96\right)$, and the margin of error (d) was set at 5%. Calculations were performed using R software version 4.4.2 based on the following formula: $n=\frac{Z_{\alpha /2}^{2}\cdot p(1-p)}{d^{2}}=\frac{1.96^{2}\cdot 0.16\cdot 0.84}{0.05^{2}}\approx 207$. Considering potential missing information in retrospective data collection and sample attrition due to matching failure during PSM, a 20% attrition rate was anticipated. Consequently, the final estimated initial sample size required was: n=207÷(1−0.20)≈259 cases. This study ultimately included 264 children with ALL who met the inclusion criteria, satisfying the requirement for the initial sample size estimation. regarding the multivariate logistic regression analysis after PSM, according to the statistical principles proposed by Riley et al. (18), a minimum of 10 outcome events per variable (EPV) is required to avoid model overfitting. In this study, the post-matching sepsis group comprised 33 cases, and the final model incorporated 3 independent variables, yielding an EPV of 11, which meets the robustness requirements for sample size in multivariate analysis.

### 2.6 Data preprocessing and missing data handling

Prior to formal statistical analysis, raw data were rigorously evaluated for missing values and outliers to ensure dataset integrity. The proportion of missing data for each variable was explicitly assessed. Demographic and disease characteristics (e.g., age, immunophenotype) and the baseline pSOFA score had completely intact data (0.00% missing). For routine complete blood count parameters (including WBC, ANC, RBC, Hb, HCT, MCV, MCH, MCHC, RDW, and PLT) and body mass index (BMI), the missing data proportion was 5.56%. Specifically, missing values were imputed using the Multiple Imputation by Chained Equations (MICE) algorithm, incorporating all relevant baseline covariates and the outcome variable into the imputation model to ensure robust estimations. Outliers were defined using the interquartile range (IQR) method as values falling below Q1-1.5 × IQR or above Q3 + 1.5 × IQR, and only verified data entry errors or implausible laboratory artifacts were excluded.

### 2.7 Statistical analysis

All statistical analyses were performed using R software version 4.4.2. Prior to analysis, raw data were cleaned to exclude missing values and outliers to ensure the integrity and accuracy of the dataset. The normality of continuous variables was assessed using the Shapiro–Wilk test. Normally distributed variables were expressed as mean ± standard deviation $\left(\bar{x}\pm s\right)$ and compared between groups using the independent samples *t*-test. Non-normally distributed variables were presented as median and interquartile range [*M (P25, P75)*] and compared using the Mann–Whitney *U* test. Categorical variables were expressed as frequencies and percentages [*n*(%)] and compared using the Chi-square test or Fisher's exact test. To effectively control for confounding bias in this observational study, Propensity Score Matching (PSM) was utilized. First, a multivariable logistic regression model was employed to calculate the propensity score for each patient. The covariates included in the propensity model were sex, number of hospitalizations, year of discharge, season of admission, and leukemia risk stratification. The rationale for selecting these variables was based on their statistically significant differences at baseline (before matching) and their potential clinical role as confounders affecting both the baseline status and the risk of infection. Subsequently, patients in the sepsis and CIN groups were matched using the nearest-neighbor matching algorithm without replacement. The maximum matching ratio was set to 1:3 (sepsis to CIN), with a strict caliper width of 0.2 standard deviations of the logit of the propensity score to ensure optimal matching quality. Post-matching analysis involved evaluating the balance of covariates using the Standardized Mean Difference (SMD), where an SMD < 0.1 was considered indicative of excellent covariate balance. All subsequent time-to-event prognostic analyses were performed exclusively on this matched cohort. Subsequently, after excluding multicollinearity using the Variance Inflation Factor (VIF), univariate and multivariate Cox proportional hazards regression models were constructed based on the post-PSM dataset to identify independent risk factors and account for the time-to-event nature of sepsis onset. The inclusion of the baseline pSOFA score as an independent predictor in the regression model was carefully considered to avoid circular reasoning. Sepsis was defined dynamically as a subsequent increase in the pSOFA score of ≥2 (△pSOFA ≥ 2) from baseline. Therefore, the baseline pSOFA score was included not as a diagnostic component, but as an essential covariate representing the patient's initial physiological reserve and pre-existing illness severity at the onset of CIN. Adjusting for this baseline static status is methodologically necessary to demonstrate the true independent predictive value of RDW and Hb for the future dynamic deterioration event.On this basis, to quantify the impact of potential unmeasured confounders on the robustness of the Cox regression results, an *E*-value sensitivity analysis was conducted. The *E*-value refers to the minimum strength of association that an unmeasured confounder would need to have with both the treatment assignment and the outcome variable to explain away the observed point estimate or the lower limit of the 95% confidence interval (i.e., reducing the hazard ratio to 1.00) (19). Previous methodological studies suggest that an *E*-value > 1.5–2.0 indicates a relatively strong resistance to unmeasured confounding. Regarding prognostic predictive performance, the discrimination of each indicator was quantified using Receiver Operating Characteristic (ROC) curves and the Area Under the Curve (AUC). The optimal cutoff values were determined based on the principle of maximizing the Youden Index. Comparison of AUCs between different indicators was performed using the DeLong test. To further assess the incremental predictive value of the combined model over single indicators, Net Reclassification Improvement (NRI) and Integrated Discrimination Improvement (IDI) were calculated. Specifically, continuous NRI was utilized to quantify the net proportion of patients whose predicted probabilities were correctly reclassified by the new model. IDI was employed to measure the overall improvement in the difference of mean predicted probabilities between the sepsis and non-sepsis groups after incorporating the combined predictors. Decision Curve Analysis (DCA) was applied to evaluate the clinical net benefit of the models. Additionally, cumulative incidence curves for sepsis were plotted based on the quartiles of RDW, Hb, and pSOFA score, and differences between groups were assessed using the Log-rank test. A *P*-value < 0.05 was considered statistically significant.

## 3 Results

### 3.1 Comparison of general information and clinical characteristics between the CIN and sepsis groups before PSM

A total of 264 children with ALL were included in this study and divided into a CIN group (*n* = 225) and a sepsis group (*n* = 39) based on whether sepsis occurred. The comparison of baseline data showed that the proportion of male patients in the sepsis group was significantly higher than that in the CIN group $\left(\chi^{2}=6.623,\;P=0.010\right)$. Regarding hospitalization characteristics and temporal distribution, statistically significant differences were observed in the frequency of hospitalizations $\left(\chi^{2}=10.644,\;P=0.031\right)$, year of discharge $\left(\chi^{2}=11.602,\;P=0.009\right)$, and season of admission $\left(\chi^{2}=18.127,\;P<0.001\right)$. Furthermore, the risk stratification in the sepsis group was significantly elevated, with high-risk patients accounting for 43.59%, showing a statistically significant difference between the groups $\left(\chi^{2}=7.626,\;P=0.022\right)$ (Table 1).

**Table 1:** Comparison of general characteristics between the two groups of children before PSM *n*(%).

Items	CIN group (*n* = 225)	sepsis group (*n* = 39)	*χ* ^2^	*P*
Number of hospitalizations			10.644	0.031
1	22 (9.78)	10 (25.64)		
2	61 (27.11)	12 (30.77)		
3	50 (22.22)	5 (12.82)		
4	56 (24.89)	5 (12.82)		
5	36 (16.00)	7 (17.95)		
Sex			6.623	0.010
Male	91 (40.44)	25 (64.10)		
Female	134 (59.56)	14 (35.90)		
Year of discharge			11.602	0.009
2021	27 (12.00)	10 (25.64)		
2022	101 (44.89)	8 (20.51)		
2023	42 (18.67)	12 (30.77)		
2024	55 (24.44)	9 (23.08)		
Season of admission			18.127	<0.001
Spring	15 (6.67)	10 (25.64)		
Summer	150 (66.67)	15 (38.46)		
Autumn	26 (11.56)	7 (17.95)		
Winter	34 (15.11)	7 (17.95)		
Risk stratification			-	0.022^a^
Standard risk	27 (12.00)	0 (0.00)		
Intermediate risk	136 (60.44)	22 (56.41)		
High risk	62 (27.56)	17 (43.59)		

^a^ Fisher's exact test.

### 3.2 Comparison of general information and clinical characteristics between the CIN and sepsis groups after PSM

To control for confounding bias between groups, PSM was utilized to balance baseline covariates. A total of 108 patients were included after matching (75 in the CIN group and 33 in the sepsis group). The results showed that there were no statistically significant differences between the two groups in baseline characteristics such as the number of hospitalizations, sex distribution, year of discharge, season of admission, and risk stratification (*P* > 0.05). Furthermore, the standardized mean difference (SMD) for all covariates was less than 0.1, indicating a well-balanced distribution of covariates between the two groups after matching (Table 2).

**Table 2:** Comparison of general characteristics between the CIN and sepsis groups after PSM *n*(%).

Items	CIN group (*n* = 75)	Sepsis group (*n* = 33)	*χ* ^2^	*P*	SMD
Number of hospitalizations			1.771	0.778	0.072
1	12 (16.00)	8 (24.24)			
2	19 (25.33)	9 (27.27)			
3	14 (18.67)	4 (12.12)			
4	15 (20.00)	5 (15.15)			
5	15 (20.00)	7 (21.21)			
Sex			0.555	0.456	<0.001
Male	38 (50.67)	20 (60.61)			
Female	37 (49.33)	13 (39.39)			
Year of discharge			1.858	0.602	0.074
2021	12 (16.00)	8 (24.24)			
2022	16 (21.33)	8 (24.24)			
2023	23 (30.67)	10 (30.30)			
2024	24 (32.00)	7 (21.21)			
Season of admission			1.216	0.749	0.067
Spring	12 (16.00)	8 (24.24)			
Summer	31 (41.33)	12 (36.36)			
Autumn	17 (22.67)	6 (18.18)			
Winter	15 (20.00)	7 (21.21)			
Risk stratification			-	0.405^a^	0.062
Standard risk	0 (0.00)	0 (0.00)			
Intermediate risk	36 (48.00)	19 (57.58)			
High risk	39 (52.00)	14 (42.42)			

^a^ Fisher's exact test.

### 3.3 Comparison of clinical characteristics and laboratory indices between the CIN and sepsis groups after PSM

Based on the post-PSM dataset, clinical characteristics and baseline laboratory indices were further compared between the two groups. The results showed no statistically significant differences in age, BMI, immunophenotype distribution, PLT, MCV, MCH, or MCHC levels between the groups (*P* > 0.05). However, the WBC (*Z* = 2.855, *P* = 0.004), ANC (*Z* = 3.441, *P* < 0.001), RBC (*Z* = 2.488, *P* = 0.013), Hb (*Z* = 2.895, *P* = 0.004), and Hct levels (*Z* = 2.254, *P* = 0.024) in the sepsis group were significantly lower than those in the CIN group. regarding the assessment of red blood cell volume heterogeneity and disease severity, the RDW (*Z* = −3.198, *P* = 0.001) and pSOFA score (*Z* = −2.337, *P* = 0.018) in the sepsis group were significantly higher than those in the CIN group (Table 3). Regarding microbiological evidence, due to the routine prophylactic and empirical use of broad-spectrum antibiotics during the CIN phase, positive blood cultures or pathogen identification were obtained in only 3 out of the 33 sepsis cases (9.1%). The identified pathogens were exclusively Gram-negative bacteria, comprising Pseudomonas aeruginosa (*n* = 2) and Klebsiella pneumoniae (*n* = 1).

**Table 3:** Comparison of clinical characteristics and laboratory indices between the CIN and sepsis groups after PSM.

Items	CIN group (*n* = 75)	sepsis group (*n* = 33)	*χ*^2^/*Z*	*P*
Age (years)	9.00 (5.97, 11.00)	8.00 (5.50, 11.00)	0.223	0.825
BMI (kg/m²)	16.26 (15.30, 17.85)	15.70 (15.22, 17.48)	0.800	0.425
Immunophenotype (*n*, %)			1.600	0.206
B-cell	59 (79)	30 (91)		
T-cell	16 (21)	3 (9)		
WBC (×10⁹/L)	0.54 (0.32, 1.33)	0.33 (0.12, 0.58)	2.855	0.004
ANC (×10⁹/L)	0.11 (0.01, 0.28)	0.01 (0.00, 0.10)	3.441	<0.001
RBC (×10⁹/L)	2.68 (2.32, 3.24)	2.35 (2.10, 2.76)	2.488	0.013
Hb (g/L)	79.00 (67.50, 100.00)	71.00 (64.00, 81.00)	2.895	0.004
Hct (%)	23.70 (20.25, 30.50)	22.40 (18.50, 24.80)	2.254	0.024
MCV (fL)	89.00 (85.60, 93.75)	90.50 (85.20, 94.10)	−0.410	0.684
MCH (pg)	29.80 (28.35, 31.30)	29.40 (28.80, 31.40)	−0.240	0.813
MCHC (g/L)	330.00 (322.50, 341.00)	332.00 (322.00, 340.00)	0.420	0.677
RDW (%)	14.80 (12.90, 15.80)	15.90 (14.60, 17.20)	−3.198	0.001
PLT (×10⁹/L)	58.00 (28.00, 119.00)	35.00 (16.00, 72.00)	1.814	0.070
pSOFA score	5 (4, 6)	5 (4, 7)	−2.337	0.018

### 3.4 Timeline of baseline sampling and subsequent sepsis diagnosis during CIN

**Figure 1:**
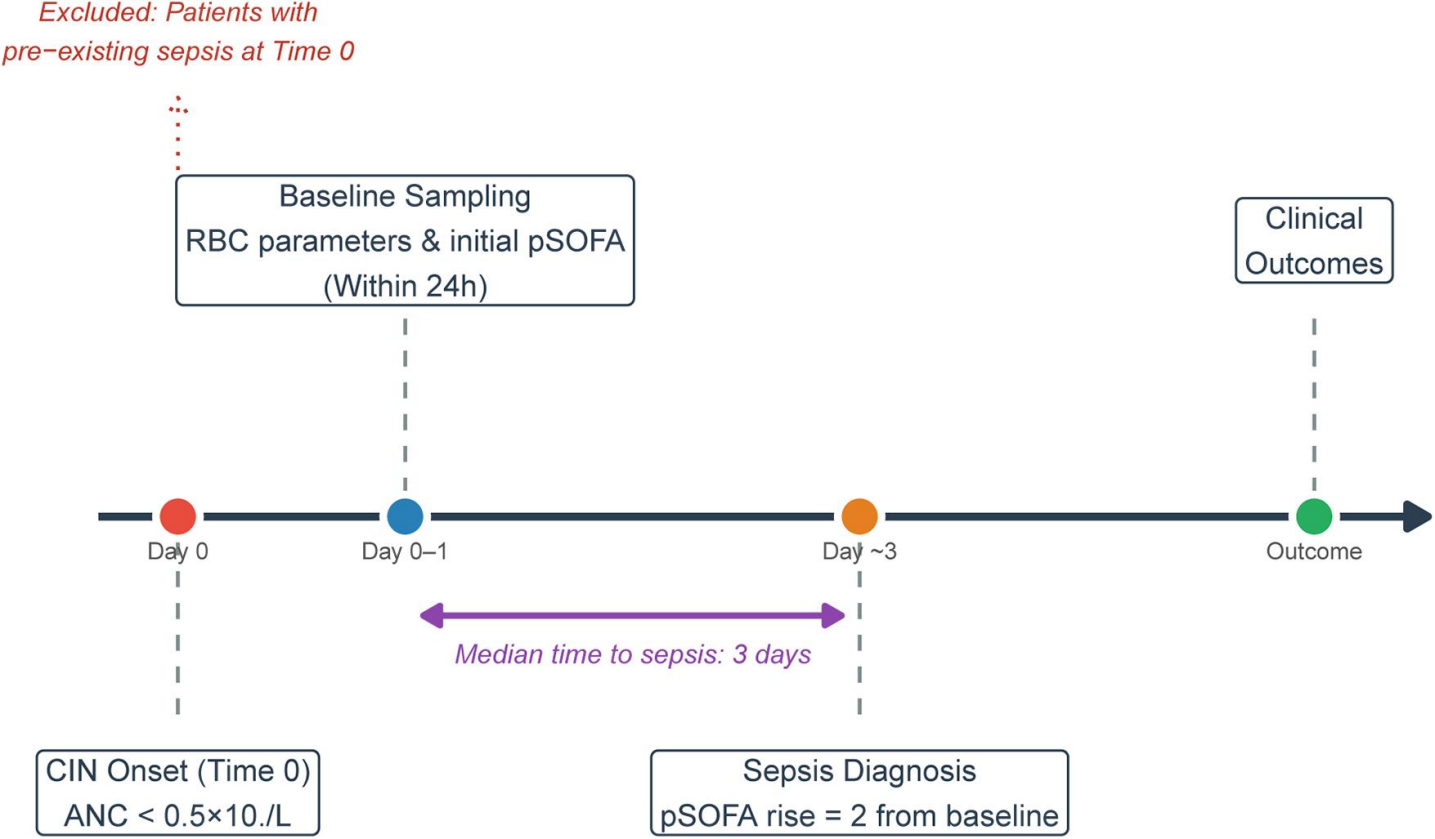
Time-anchoring flowchart of the study design. The timeline delineates the temporal sequence from the onset of chemotherapy-induced neutropenia (CIN, defined as absolute neutrophil count <0.5 × 10^9^/L at Time 0), to the baseline clinical and laboratory assessments (within 24 h), and the subsequent continuous monitoring for the clinical diagnosis of sepsis [defined as a pediatric Sequential Organ Failure Assessment (pSOFA) score increase ≥2 from baseline] in children with ALL. Patients with pre-existing sepsis at Time 0 were excluded to prevent incorporation bias.

To ensure the true predictive validity of the biomarkers, a clear time-anchoring approach was strictly followed. The onset of CIN was defined as time zero (T0). Baseline organ function data for the initial pSOFA score and blood samples for RBC parameters were synchronously collected within 24 h of T0. Patients presenting with sepsis at T_0 were excluded. In the final matched cohort, the median time from CIN onset to the diagnosis of sepsis was 3 days (IQR: 1–6 days). Specifically, 45.5% of the sepsis cases were diagnosed within 48 h, while 54.5% were diagnosed beyond 48 h after baseline sampling. This temporal separation confirms that the baseline RDW and Hb levels were evaluated prior to the clinical onset of subsequent septic events, mitigating the risk of incorporation bias (Figure 1).

### 3.5 Cox proportional hazards regression analysis of sepsis occurrence during CIN in children with ALL based on PSM

A univariate Cox proportional hazards regression model was employed to analyze potential factors influencing the time to sepsis occurrence during CIN in children with ALL. The results indicated that RDW (*HR* = 1.204, 95% *CI*: 1.118–1.298, *P* < 0.001) and pSOFA score (*HR* = 1.422, 95% *CI*: 1.127–1.796, *P* = 0.003) were risk factors for sepsis, whereas Hb (*HR* = 0.979, 95% *CI*: 0.962–0.997, *P* = 0.020) was a protective factor. Subsequently, the statistically significant variables (Hb, RDW, and pSOFA score) were included in a multivariate Cox regression model. The time-to-event analysis revealed that baseline RDW (*HR* = 1.175, 95% *CI*: 1.088–1.270, *P* < 0.001) and pSOFA score (*HR* = 1.297, 95% *CI*: 1.006–1.673, *P* = 0.045) remained independent temporal predictors for the future onset of sepsis during CIN. Furthermore, *E*-value sensitivity analysis showed that the E-values for the HR of RDW and pSOFA score were 1.629 and 1.918, respectively. The *E*-values for the lower confidence interval limits of both variables were also greater than 1 (1.397 and 1.081, respectively), suggesting that the temporal associations between these independent risk factors and sepsis are robust (Tables 4, 5).

**Table 4:** Univariate Cox proportional hazards regression analysis of sepsis occurrence during CIN in children with ALL based on PSM.

Items	*β*	*SE*	Wald*χ*2	*P*	*HR*(95% *CI*)	*E*-value for OR	*E*-value for lower CI of OR
WBC	−0.026	0.062	0.171	0.679	0.975 (0.862–1.101)	-	-
RBC	0.002	0.011	0.036	0.849	1.002 (0.981–1.023)	-	-
Hb	−0.021	0.009	5.437	0.020	0.979 (0.962–0.997)	1.168	1.061
Hct	−0.018	0.021	0.715	0.398	0.983 (0.943–1.024)	-	-
RDW	0.186	0.038	23.851	<0.001	1.204 (1.118–1.298)	1.700	1.480
pSOFA	0.352	0.119	8.765	0.003	1.422 (1.127–1.796)	2.198	1.504

**Table 5:** Multivariate Cox proportional hazards regression analysis of sepsis occurrence during CIN in children with ALL based on PSM.

Items	*β*	*SE*	Wald*χ*2	*P*	*HR*(95% *CI*)	*E*-value for OR	*E*-value for lower CI of OR
Hb	−0.007	0.010	0.556	0.456	0.993 (0.973–1.012)	-	-
RDW	0.161	0.039	16.782	<0.001	1.175 (1.088–1.270)	1.629	1.397
pSOFA	0.260	0.130	4.015	0.045	1.297 (1.006–1.673)	1.918	1.081

### 3.6 Predictive efficacy and incremental value analysis of different indicators for sepsis during CIN in children with ALL based on PSM

**Figure 2:**
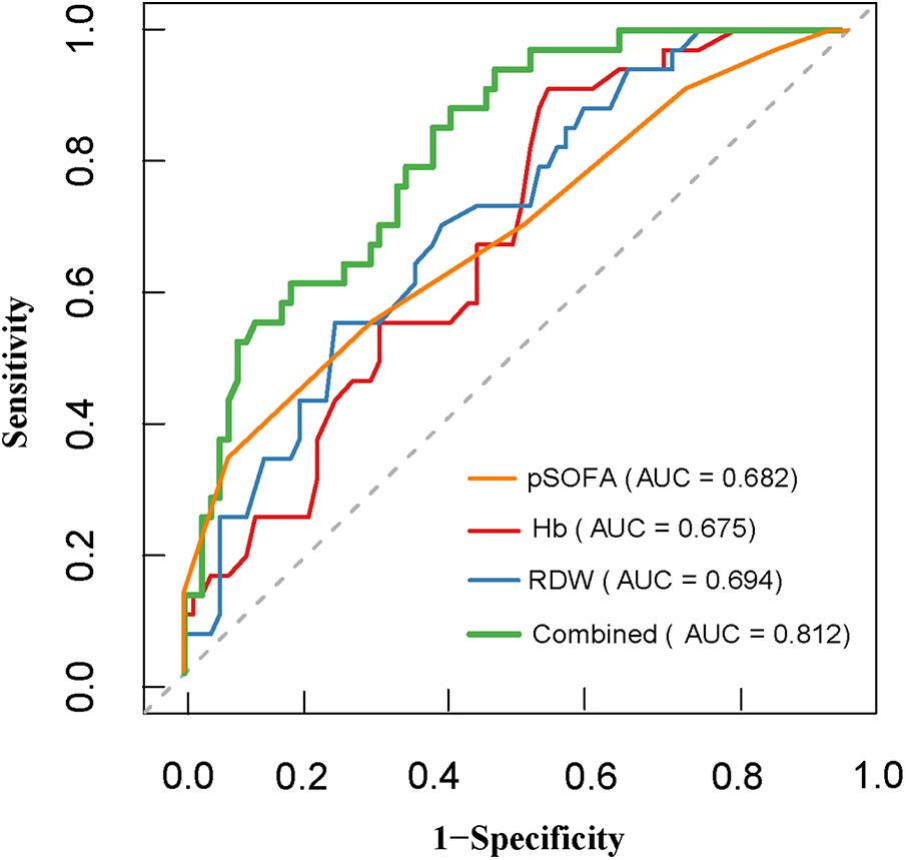
Receiver operating characteristic (ROC) curves of different clinical indicators for predicting sepsis occurrence during CIN in children with ALL. The predictive efficacy of Hb, RDW, pSOFA score, and their combined model (Combined: Hb + RDW) were evaluated. The combined model exhibited the highest area under the curve (AUC = 0.812), indicating superior discrimination capacity for early sepsis prediction compared to any single indicator. ALL, acute lymphoblastic leukemia; CIN, chemotherapy-induced neutropenia.

The predictive efficacy of Hb, RDW, pSOFA score, and the combined model for sepsis during CIN in children with ALL was evaluated using ROC curves (0 = non-occurrence, 1 = occurrence). The results showed that among the single indicators, RDW demonstrated relatively high predictive efficacy, with an AUC of 0.701 (95% *CI*: 0.598–0.804). When the optimal cutoff value was set at 15.850, the specificity was 0.770 and the sensitivity was 0.546. The AUCs for Hb and pSOFA score were 0.672 (95% *CI*: 0.568–0.777) and 0.682 (95% *CI*: 0.570–0.795), respectively. To further improve predictive efficacy, a combined predictive indicator (Combined) incorporating Hb and RDW was constructed. Analysis indicated that the AUC of the Combined model increased to 0.812 (95% *CI*: 0.730–0.895). DeLong's test results demonstrated that the predictive efficacy of the Combined model was significantly superior to that of Hb (*P* < 0.001) and RDW (*P* = 0.003). Furthermore, NRI and IDI were employed to evaluate the incremental predictive value of the combined model. The analysis results showed that compared to the single indicator RDW, the combined model exhibited significant integrated discrimination improvement benefits, with an IDI of 0.068 (95% *CI*: 0.024–0.114, *P* = 0.003) (Tables 6, 7 and Figure 2).

**Table 6:** Evaluation of diagnostic efficacy of ROC curves for different clinical indicators in predicting sepsis during CIN in children with ALL.

Items	AUC (95% *CI*)	Optimal cutoff value	Sensitivity	Specificity	PPV	NPV	Accuracy	Youden Index	*P*
Hb	0.672 (0.568–0.777)	84.500	0.909	0.446	0.423	0.917	0.589	0.355	<0.001
RDW	0.701 (0.598–0.804)	15.850	0.546	0.770	0.514	0.792	0.701	0.316	0.003
pSOFA	0.682 (0.570–0.795)	6.500	0.333	0.932	0.688	0.758	0.748	0.266	0.059
Combined	0.812 (0.730–0.895)	0.253	0.879	0.595	0.492	0.917	0.682	0.473	-

Combined refers to the joint predictive indicator containing Hb and RDW; PPV, positive predictive value; NPV, negative predictive value. *P* represents the comparison result of the AUC of the Combined model with that of each single indicator using DeLong's test.

**Table 7:** Incremental value analysis of the joint predictive indicator for sepsis prognosis in children with ALL.

Items	NRI	NRI (95% *CI*)	NRI *P*	IDI	IDI (95%*CI*)	IDI *P*
Combined vs. Hb	−0.126	(−0.529, 0.269)	0.540	0.000	(−0.001, 0.001)	0.680
Combined vs. RDW	0.313	(−0.090, 0.703)	0.122	0.068	(0.024, 0.114)	0.003
Combined vs. pSOFA	0.010	(−0.400, 0.412)	0.962	−0.024	(−0.093, 0.046)	0.492

Combined refers to the joint predictive indicator containing Hb and RDW.

### 3.7 Clinical benefit analysis of the combined predictive indicator for sepsis during CIN in children with ALL based on PSM

**Figure 3:**
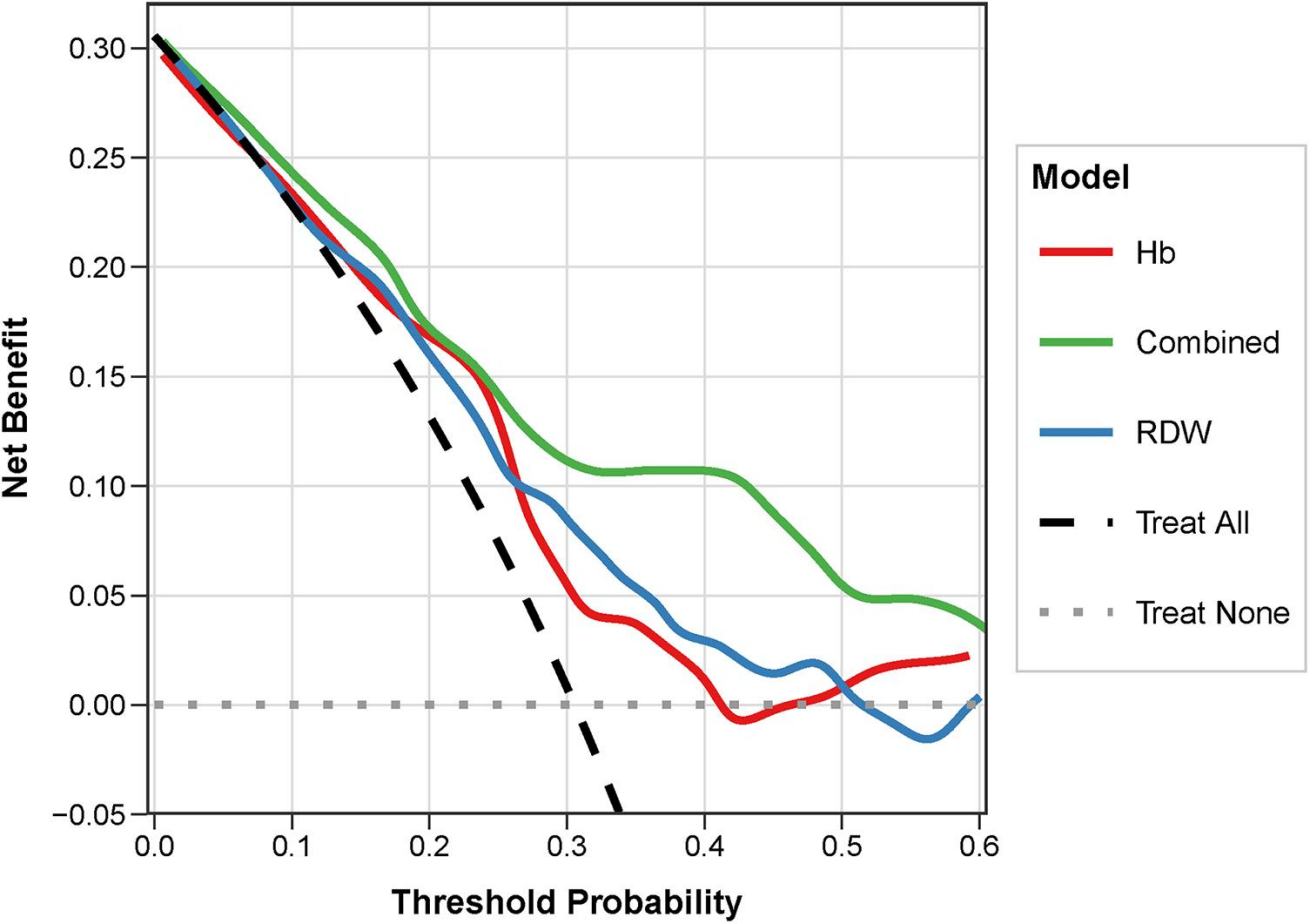
Decision curve analysis (DCA) of different clinical indicators for predicting sepsis occurrence during CIN in children with ALL. The *x*-axis represents the threshold probability, and the *y*-axis represents the net clinical benefit. The black horizontal line indicates the assumption that no patient develops sepsis (net benefit = 0), and the grey line assumes all patients develop sepsis. The red line representing the Combined model (Hb + RDW) remains the highest across a broad range of threshold probabilities (10%–60%), indicating that utilizing the combined model for clinical decision-making provides a greater net clinical benefit and utility than relying on single indices. ALL, acute lymphoblastic leukemia; CIN, chemotherapy-induced neutropenia.

To further evaluate the application value of the Combined indicator in clinical practice, DCA curves were constructed. The DCA results showed that within the threshold probability range of approximately 10% to 60%, the net benefit of the Combined indicator was generally superior to that of the single indicators Hb and RDW, demonstrating superior clinical utility and early warning efficacy (Figure 3).

### 3.8 Analysis of cumulative incidence of sepsis based on quartiles of clinical indicators

**Figure 4:**
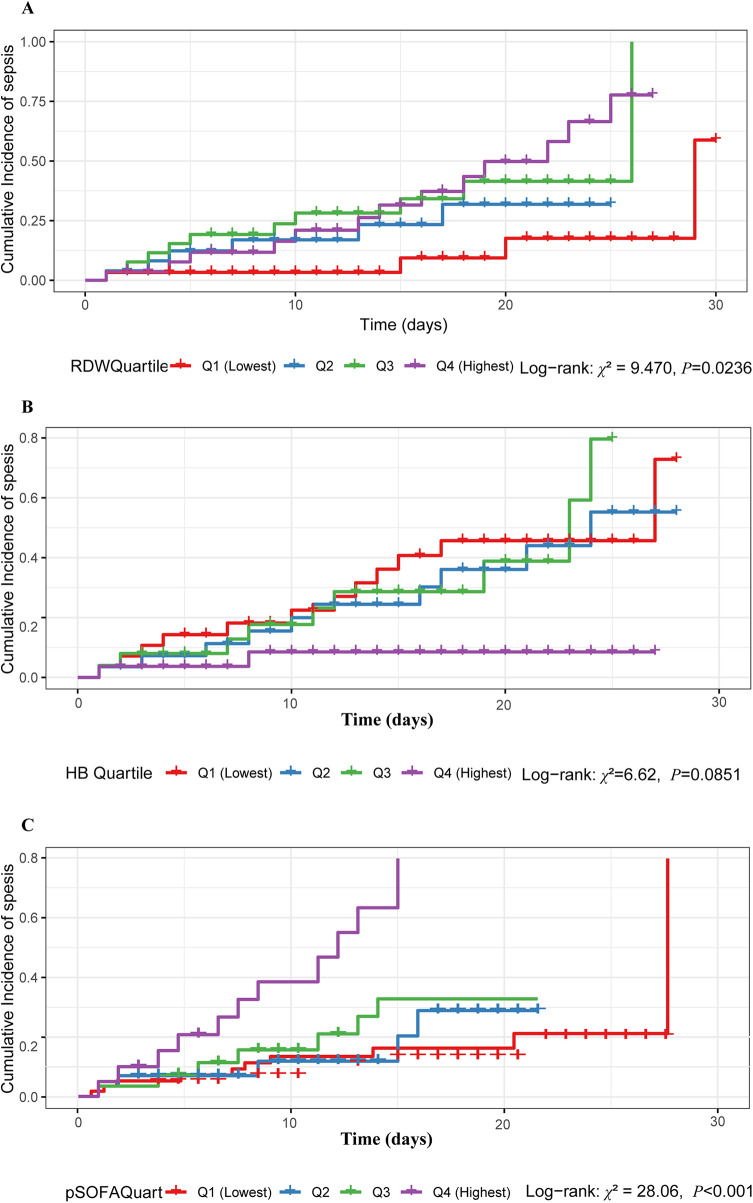
Kaplan-Meier cumulative incidence curves of sepsis during CIN in children with ALL, stratified by quartiles of baseline clinical indicators. **(A)** Stratification by RDW quartiles shows a significant time-dependent risk gradient. **(B)** Stratification by Hb quartiles did not reach statistical significance. **(C)** Stratification by pSOFA score quartiles demonstrated significant risk discrimination. These results indicate that RDW and pSOFA are robust standalone indices for dynamic, time-dependent risk stratification. ALL, acute lymphoblastic leukemia; CIN, chemotherapy-induced neutropenia; RDW, red blood cell distribution width; Hb, hemoglobin; pSOFA, pediatric Sequential Organ Failure Assessment.

To further evaluate the time-dependent impact of clinical indicators on the occurrence of sepsis, children with ALL were stratified into four quartiles (Q1–Q4) based on baseline RDW, Hb, and pSOFA levels, and cumulative incidence curves for sepsis were plotted. The Log-rank test results indicated that the differences in the cumulative incidence of sepsis among the different RDW level groups (*χ*2 = 9.470, *P* = 0.024) and pSOFA score groups (*χ*^2^ = 28.06, *P* < 0.001) were statistically significant (Figures 4A,C). However, the cumulative incidence across Hb quartiles did not reach statistical significance (*χ*^2^ = 6.62, *P* = 0.085) (Figure 4B).

## 4 Discussion

This study focused on the clinical limitation of restricted early recognition of sepsis in children with ALL during chemotherapy-induced CIN. By applying PSM to effectively correct for the confounding interference of chemotherapy heterogeneity and baseline characteristics, we confirmed that elevated RDW accompanied by decreased Hb serves as an independent early warning signal for the occurrence of sepsis. This finding suggests that red blood cell parameters not only reflect bone marrow hematopoietic function but also act as a dual monitor of inflammation and metabolism independent of the leukocyte system, helping to compensate for the reduced sensitivity of conventional infection markers during the myelosuppression phase.

The independent predictive efficacy of RDW highlights the pathophysiological changes in erythropoiesis kinetics under acute inflammation. Previous studies have shown that in the early stages of sepsis, pathogen-associated molecular patterns activate Toll-like receptors, inducing the mononuclear phagocyte system to release pro-inflammatory mediators such as tumor necrosis factor-α (TNF-α) and IL-6. This process downregulates the expression of the key erythroid transcription factor GATA-1 binding protein via the activation of the nuclear factor kappa-B (NF-κB) signaling pathway, directly blocking the erythroid-specific transcriptional network and anti-apoptotic signaling pathways, thereby inhibiting the proliferation and differentiation of erythroid progenitors (20). Nemeth et al. found that IL-6 induces ferritin degradation and inhibits ferroportin by upregulating hepatic hepcidin synthesis, leading to functional iron deficiency and the loss of volume uniformity in newly generated red blood cells (21). Additionally, the systemic oxidative stress triggered by sepsis causes membrane lipid peroxidation and cross-linking rupture of cytoskeletal proteins, severely impairing red blood cell deformability and shortening their lifespan. This forces the bone marrow to compensatorily release large, immature reticulocytes, further exacerbating the heterogeneity of peripheral red blood cell volume (22). Regarding the early warning value of Hb, our results align with the mechanism of anemia of inflammation elucidated by Weiss et al. (11). The hypermetabolic state of sepsis sharply increases the body's oxygen consumption, leading to the redistribution of microcirculatory blood flow and prioritizing damage to the intestinal mucosal barrier, which is extremely sensitive to hypoxia. Persistent tissue hypoxia induces the abnormal accumulation of hypoxia-inducible factor-1*α* (HIF-1*α*) in intestinal epithelial cells, which subsequently downregulates the expression of tight junction proteins such as Zonula occludens-1 (ZO-1), resulting in a pathological increase in intestinal permeability (23). This disruption of molecular structure promotes the translocation of endogenous intestinal bacteria and endotoxins into the blood, triggering a secondary inflammatory hit and ultimately forming a vicious cycle of anemia-hypoxia-barrier failure-sepsis progression.

Further ROC curve analysis showed that the Combined model constructed from Hb and RDW possessed superior diagnostic efficacy compared to single indicators and the pSOFA score. Duff et al., in their latest study on sepsis biomarker kinetics, pointed out that single CRP indicators often lack sensitivity in the early stages of infection due to the delayed hepatic synthesis response (peak lag of 24–48 h) (24). Addressing the limitations of single indicators in early infection, this study introduced NRI and IDI metrics to quantitatively evaluate the risk reclassification improvement ability of the combined model. The analysis confirmed that the inclusion of Hb and RDW effectively corrected the prediction bias of single indicators, primarily by correctly classifying those occult sepsis patients with low inflammatory responses (i.e., CRP not yet significantly elevated) into the high-risk group, thereby significantly enhancing the overall sensitivity of the model. Furthermore, compared to the pSOFA score proposed by Seymour et al. in the Sepsis-3 definition, which mainly relies on clinical signs following organ failure such as hypotension and altered consciousness and thus has significant lag (25), abnormal fluctuations in red blood cell parameters essentially capture the cumulative biological damage of the body in the subclinical phase. This allows for the integration and presentation of microscopic signals of impaired cell membrane rheology and ineffective hematopoiesis before macro-hemodynamics collapse. Further DCA decision curve analysis showed that within the threshold probability range of 10%–60%, the Combined model provided a net benefit significantly superior to single indicators. In terms of clinical stratification application, Yu et al. reported a positive correlation between RDW quartiles and mortality risk in children with sepsis (10). Our study extends this conclusion to the field of early diagnosis. Based on the temporal risk characteristics revealed by Kaplan–Meier survival analysis, we suggest that a dynamic stratification management mechanism based on red blood cell parameters should be established in clinical practice: for children in the high RDW group (Q4), even during a window period with stable vital signs, they should be defined as a high-risk subgroup for occult sepsis, and monitoring intervals should be shortened to achieve early warning and intervention for microcirculatory failure.

In summary, red blood cell parameters not only reflect hematopoietic function but also serve as sensitive surrogate markers for early microcirculatory dysfunction in children with ALL during the CIN phase. The combined model constructed in this study statistically confirmed its identification efficacy, making risk interception possible in the subclinical phase before hemodynamic collapse. However, this study still has certain limitations. As a single-center retrospective analysis, despite optimization using PSM, the influence of potential confounding factors such as selection bias and home care environments cannot be completely eliminated. Secondly, due to the extremely low positive rate of blood cultures (<10%) commonly observed in patients receiving prophylactic antibiotics, the sample size of culture-positive cases was statistically insufficient. Therefore, subgroup analyses for specific pathogens (such as Gram-negative or Gram-positive bacteria) were not performed, preventing the elucidation of potential pathogen-specific differences in RBC parameters.Additionally, in retrospective data, the myelotoxicity of high-intensity chemotherapy drugs themselves is difficult to completely disentangle from hematopoiesis suppression caused by infection. More importantly, the diagnostic criteria for sepsis in our study relied on an acute increase in the pSOFA score of ≥2, which aligns with the pediatric adaptation of the Sepsis-3 consensus. However, in this specific cohort, acute organ dysfunction is frequently driven by non-infectious etiologies, such as severe chemotherapy toxicity, substantial fluid shifts during hydration, or hemodynamic instability secondary to thrombocytopenia-related bleeding. These confounding factors can independently elevate the pSOFA score in the absence of an infectious response, thereby potentially introducing misclassification bias. This misclassification theoretically suggests that the robust predictive value of RDW and Hb observed in our study might not only reflect infectious pathways but could also represent a generalized systemic vulnerability and a state of cumulative physiological exhaustion in the patients. Therefore, future research urgently needs to verify the biological robustness and clinical generalizability of this predictive system through multi-center prospective cohorts, develop modified oncology-specific organ dysfunction scoring systems, and conduct mechanistic studies combined with flow cytometry.

## 5 Conclusion

RBC parameters (RDW, Hb) are independent predictors of sepsis in children with ALL during CIN. The combined prediction model constructed by Hb and RDW can effectively capture early signals of microcirculatory dysfunction, with predictive efficacy and clinical benefit superior to single indicators.

## Data Availability

The raw data supporting the conclusions of this article will be made available by the authors, without undue reservation.
